# Xuebijing injection reduces organ injuries and improves survival by attenuating inflammatory responses and endothelial injury in heatstroke mice

**DOI:** 10.1186/s12906-015-0519-5

**Published:** 2015-02-05

**Authors:** Qiulin Xu, Jingxian Liu, Xiaohua Guo, Youqing Tang, Gengbiao Zhou, Yanan Liu, Qiaobing Huang, Yan Geng, Zhifeng Liu, Lei Su

**Affiliations:** Department of ICU, General Hospital of Guangzhou Military Command, Key Laboratory of Tropical Zone Trauma Care and Tissue Repair of PLA, Guangzhou, 510010 China; Postdoctoral Workstation, Huabo Bio-pharmaceutical Research Institute, Guangzhou, 510515 China; Department of Pathophysiology, Southern Medical University, Key lab of Shock and Microcirculation Research of Guangdong Province, Guangzhou, 510515 China; Guangzhou University of Chinese Medicine, Guangzhou, 510405 China; Southern Medical University, Guangzhou, 510515 China

**Keywords:** Xuebijing injection, Heatstroke, Inflammatory responses, Endothelial injury

## Abstract

**Background:**

The pathogenesis of heatstroke is a multi-factorial process involved with an interplay among subsequent inflammation, endothelial injury and coagulation disturbances, which makes pharmacological therapy of heatstroke a challenging problem. Xuebijing injection (XBJ), a traditional Chinese medicine used to sepsis, has been reported to suppress inflammatory responses and restore coagulation disturbances. However, little is known about the role of XBJ in heatstroke.

**Methods:**

Mice were treated with indicated dose of XBJ before and/or after the induction of heatstroke. Serum inflammatory cytokines, tumor necrosis factor-α (TNF-α) and interleukin-6 (IL-6), and endothelial markers, von Willebrand Factor (vWF) and E-selectin, were measured by ELISA. Liver, kidney and heart profiles including alanine aminotransferase, aspartic aminotransferase, creatinine, blood urea nitrogen, and lactate dehydrogenase, were evaluated by UniCel DxC 800 Synchron Clinical Systems, and troponin was measured by ELISA. Coagulation profiles, including thrombin time, prothrombin time, activated partial thromboplastin time, international normalized ratio, and fibrinogen were examined by STA Compact® Hemostasis System. Jejunum injury was evaluated with H&E staining. Changes in mitochondrial structure in cardiac tissue were assesed by electron microscopy.

**Results:**

Pretreatment with XBJ decreased serum pro-inflammatory cytokines including TNF-α and IL-6, as well as endothelial injury markers, vWF and E-selectin, in a dose-dependent manner in heatstroke mice. Similar protective effects were observed when XBJ was administered after, or both before and after heat insult. These protective effects lasted for over 12 h in mice receiving XBJ before and after heat insult. XBJ also improved survival rates in heatstroke mice, ameliorated liver, heart, and kidney injuries, including mitochondrial damage to the heart, and reduced coagulation disturbances.

**Conclusions:**

XBJ prevents organ injuries and improves survival in heatstroke mice by attenuating inflammatory responses and endothelial injury. XBJ may be a potentially useful in the prevention and treatment of heatstroke.

## Background

Heatstroke, a potentially fatal condition occurring during summer heat waves, is characterized by core body temperature as high as 42°C accompanied by central nervous system dysfunction. Despite advances in prevention and treatment, the mortality rate from heatstroke remains high, varying from 10% to 50% [1]. Severe heatstroke is often accompanied by multiple organ dysfunction syndrome (MODS), which is resulted from the complex interplay among endothelial injury, inflammation, and coagulation responses of the host following the direct cytotoxic effects of heat [2]. Heat stress increases vascular permeability, promotes a pro-thrombotic state, and enhances the in vitro expression of adhesion molecules in endothelium. Markedly elevation in the concentrations of circulating inflammatory cytokines, including interleukin-6 (IL-6), tumor necrosis factor-α (TNF-α), and interleukin-1β (IL-1β), have been observed and histological examination has shown widespread hemorrhage, thrombosis, and migration of leukocytes both in animals and patients with severe heatstroke [3-7]. This constellation of events alters blood flow in the microcirculation, resulting in injuries to the endothelium and tissues. Although several drugs have been found to reduce inflammatory responses and improve prognosis in animals with heatstroke [8-13], none has been shown to reduce mortality in patients with heatstroke.

Xuebijing (XBJ) is a traditional Chinese combination medicine, which has been approved by the State Food and Drug Administration of China for the treatment of sepsis. XBJ consists mainly of extracts from five Chinese herbals: *Carthamus tinctorius L.*, *Paeonia lacti-flora Pall*, *Ligusticum chuanxiong hort*, *Salvia miltiorrhiza Bunge*, and *Angelica sinensis*. Injected XBJ has been widely used to treat sepsis-related illnesses, including acute liver and kidney injury and autoimmune diseases [14-16]. XBJ was also found to reduce circulating TNF-α, IL-6, IL-1β, and IL-8 [17,18], to maintain immunologic balance by correcting disorders of T-lymphocyte subpopulations, and to alleviate endotoxin-induced disseminated intravascular coagulation in rabbits [19]. We recently reported that XBJ alleviated liver injury in heatstroke rats [16]. However, the role and underlying mechanisms of XBJ in heatstroke remain to be determined. This study was designed to assess the role of XBJ in the release of inflammatory cytokines, in coagulation abnormalities and in endothelial injury in heatstroke mice.

## Methods

### Chemicals and reagents

XBJ, consisting of a 1:1:1:1:1 mixture of extracts of five herbs, *Carthamus tinctorius L.*, *Paeonia lacti-flora Pall*, *Ligusticum chuanxiong hort*, *Salvia miltiorrhiza Bunge*, and *Angelica sinensis*, was supplied by Tianjin Chase Sun Pharmaceutical Co., Ltd (Tianjin, China; batch number of 1303031), and stored at room temperature.

### Animals treatments

All protocols were approved by the Animal Ethics Committee of the General Hospital of Guangzhou Military Command, in accordance with the Guide for the Care and Use of Laboratory Animals of the National Institutes of Health. Male C57BL/6 J mice, aged 10–12 weeks and weighing 20–25 g, were obtained from the Animal Resource Center of Southern Medical University and maintained at 23 ± 1°C with a 12-hour light/dark cycle. Animals were randomized into three groups, a sham-heated group (Sham), a heatstroke group (HS) and a heatstroke plus XBJ group (HSXBJ). Four animals in each group were assessed for biomarkers at each indicated time point, with survival assessed in eight mice in the Sham group and 22 each in the HS and HSXBJ groups. Animals were intraperitoneally injected with 0.5 ml of 0.9% saline or the indicated doses of XBJ dilutions at the indicated times. XBJ doses were based on the doses used in patients with sepsis, with 1 ml/kg used to treat moderate sepsis and 2 ml/kg used to treat severe sepsis plus MODS. Heatstroke was induced by placing the animals in an artificial climate chamber at 38 ± 0.5°C and a relative humidity of 60% ± 5%. Rectal temperature (Tc) was measured every 10 min by a thermometer. When Tc reached 42.7°C, the criterion for heatstroke onset, the animals were removed from the chamber and allowed to recover at room temperature (24 ± 2°C). Animals in the Sham group were treated identically to those in the HS group, except that artificial climate chamber was maintained at 24 ± 0.5°C. Animals were anesthetized by intraperitoneal injection of sodium pentobarbital (50 mg/kg body weight) and sacrificed at the indicated times after heatstroke onset. Bloood and organ samples were then collected. Serum concentrations of cytokines and biochemical markers, as well as histological and ultramicrostructural changes, were assessed in animals sacrificed 6 h after heatstroke onset. To determine the time-course of serum IL-6, TNF-α, vWF, and E-selectin concentrations after heatstroke induction, animals were sacrificed at 0, 2, 4, 6, 8, 10, and 12 h after heatstroke induction. Animals used to evaluate survival rate were from separated group and were not sacrificed until 72 h of observation.

### Enzyme-linked immunosorbent assay (ELISA)

Blood samples were collected by cardiac puncture, maintained at room temperature for 30 min, and centrifuged at 1300 × g for 15 min to obtain serum. Serum samples were stored in aliquots at -80°C until assayed. The serum concentrations of TNF-α, IL-6, von Willebrand Factor antigen (vWF:Ag) (Ramco Laboratory) and E-selectin were evaluated by ELISA (R & D Systems) according to the manufacturer’s instructions .

### Organ injuries

Liver, kidney and heart profiles, including alanine aminotransferase (ALT), aspartic aminotransferase (AST), creatinine (Cr), blood urea nitrogen (BUN), and lactate dehydrogenase (LDH) levels, were evaluated by UniCel DxC 800 Synchron Clinical Systems (United States), and troponin (Tn) concentration was determined by ELISA (MyBioSource, United States). Coagulation profiles including thrombin time (TT), prothrombin time (PT), activated partial thromboplastin time (APTT), international normalized ratio (INR), and fibrinogen concentration, were assessed using an STA Compact® Hemostasis System (France).

### Histological examination

Tissue samples from jejunum were obtained 6 h after heatstroke onset, fixed in 4% paraformaldehyde, embedded in paraffin, cut into 5-μm sections with a microtome, and stained with hematoxylin and eosin. Stained specimens were examined by light microscopy.

### Electron microscopy

Cardiac tissue was quickly cut into 1-mm cubes, incubated overnight at 4°C in 2.5% glutaraldehyde in 0.1 mol/l phosphate buffer (pH 7.4), and fixed in 1% buffered osmium tetroxide. The specimens were conventionally processed and evaluated by electron microscopy (H-800; Hitachi).

### Statistical analysis

All continuous data were expressed as mean ± SE and compared by one-way analysis of variance with Fisher's LSD test. Survival curves were plotted by the Kaplan-Meier method and compared by the log-rank test. P < 0.05 was considered statistically significant. All statistical analyses of data were performed using SPSS 16.0 (SPSS Inc., Chicago, IL).

## Results

### Dose-dependent effects of XBJ on inflammation and endothelial injury

**Figure 1:**
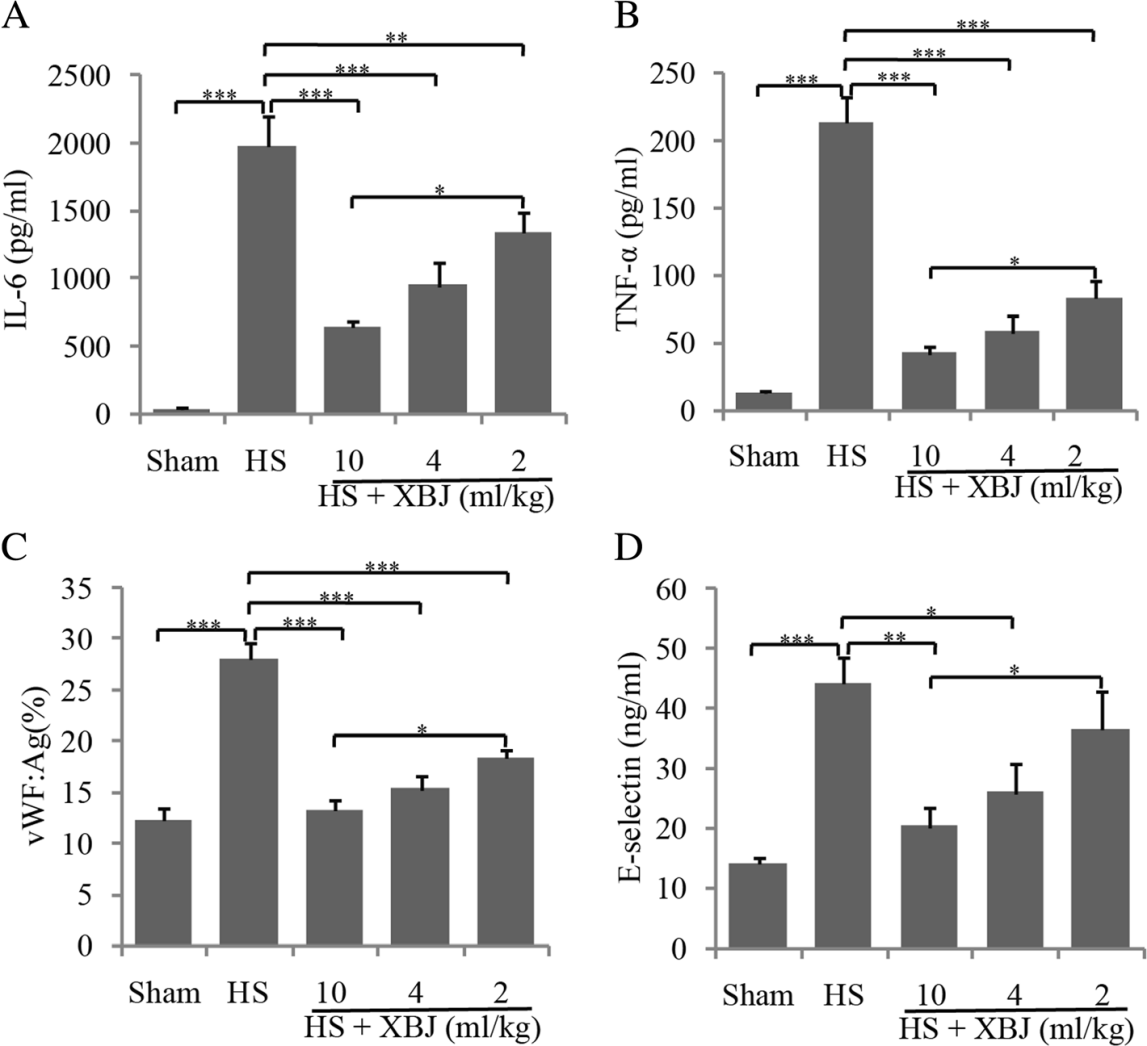
**Dose-dependent effects of XBJ on serum IL-6, TNF-α, vWF and E-selectin.** Mice were treated with 0.9% saline or 2, 4, or 10 ml/kg of XBJ, followed by heat insult. Serum IL-6 **(A)**, TNF-α **(B)**, vWF **(C)**, and E-selectin **(D)** levels were determined with ELISA 6 h after heatstroke onset. *P < 0.05, **P < 0.01, ***P < 0.001.

E-selectin and vWF, regarded as biomarkers of endothelial injury, and IL-6 and TNF-α, key factors in systemic inflammatory responses, have been reported to be increased in heatstroke [1,3]. To determine the role of XBJ in heatstroke-induced inflammatory responses and endothelial injury, we assessed whether XBJ affects serum levels of these four proteins in heatstroke. Mice were treated with 2, 4, or 10 ml/kg of XBJ before heat insult, and serum concentrations of IL-6, TNF-α, E-selectin and vWF were measured 6 h after heatstroke onset. The concentrations of all four proteins were higher in the HS than in the Sham group. XBJ dose-dependently reduced the serum concentrations of these four proteins, with 10 mg/kg of XBJ reducing serum IL-6 and TNF-α about 80% each and serum E-selectin and vWF about 50% each (Figure 1A-D). As 2 ml/kg of XBJ is used to treat septic patients with MODS, further experiments assessed the effects of 4 ml/kg of XBJ, thus balancing experimental effects and clinical significance.

### Effects of time of XBJ administration on inflammation and endothelial injury

**Figure 2:**
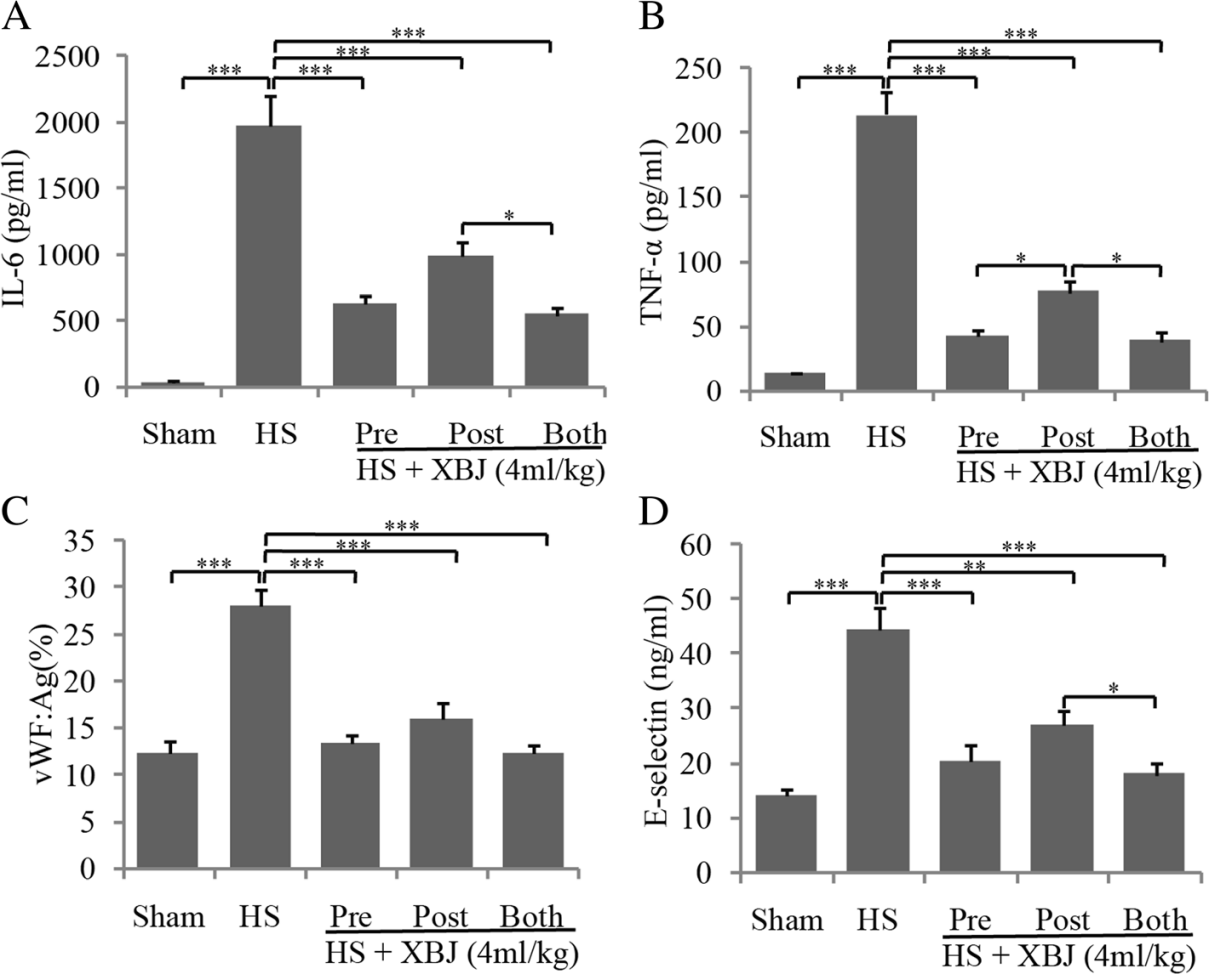
**Effects of administration time of XBJ on serum IL-6, TNF-α, vWF and E-selectin.** Mice subjected to heat stress were treated with 0.9% saline or 4 ml/kg of XBJ 30 min before heat insults (Pre), post heatstroke onset (Post), or both before heat insults and post heatstroke onset (Both). Serum IL-6 **(A)**, TNF-α **(B)**, vWF **(C)**, and E-selectin **(D)** levels were determined with ELISA 6 h after heatstroke onset. *P < 0.05, **P < 0.01, ***P < 0.001.

To investigate whether the effects of XBJ administered after heatstroke onset were similar to those of XBJ administered before heat insult, mice were treated with the same dose of XBJ before or after heatstroke. Interestingly, we found the XBJ-induced reductions in IL-6, E-selectin and vWF concentrations were similar in mice treated with XBJ before heat insult or after heatstroke onset, whereas serum TNF-α was lower in in mice treated with XBJ before heat insult than after heatstroke onset. As a traditional Chinese medicine compound, the effective duration of XBJ is difficult to determine and not reported. The recommended time interval of consecutive uses of XBJ is usually 12 h, and 6 to 8 h for critical patients according to its clinical instructions. In studies with human or animals, XBJ is usually used 1 to 3 times per day. In this set, we administered mice a second dose of XBJ after heatstroke since the experiment spanned 5-h of heat insult and 6-h of observation. However, the second dose of XBJ did not result in additional decreases in these four proteins compared with the same dose of saline given after heatstroke, but did reduce serum concentrations of IL-6, TNF-α, and E-selectin (Figure 2A-D). These findings suggested that XBJ attenuated inflammatory responses and endothelial injury when given after heatstroke.

### Influences of XBJ on time course of inflammatory and endothelial biomarkers

**Figure 3:**
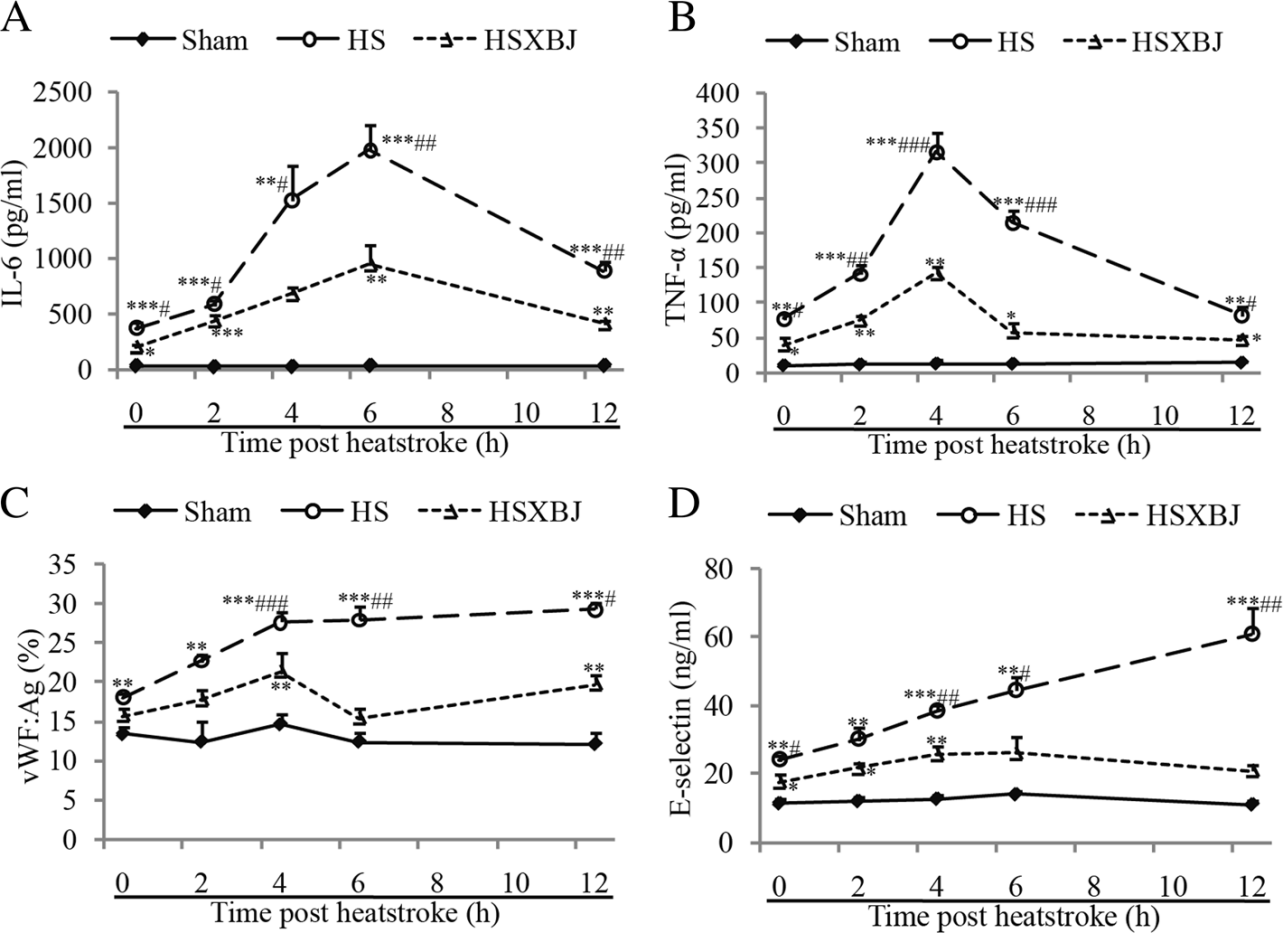
**Time course of serum IL-6, TNF-α, vWF, and E-selectin after heatstroke induction.** Mice were treated with 0.9% saline (HS) or 4 ml/kg of XBJ (HSXBJ), followed by heat insult. Serum IL-6 **(A)**, TNF-α **(B)**, vWF **(C)**, and E-selectin **(D)** levels were determined with ELISA at 0, 2, 4, 6, and 12 h after heatstroke onset. *P < 0.05, **P < 0.01, ***P < 0.001, vs. sham group; ^#^P < 0.05, ^##^P < 0.01, ^###^P < 0.001, vs. HSXBJ group.

To further assess the effects of XBJ on pro-inflammatory cytokines and markers of endothelial injury, we analyzed the influences of XBJ on the kinetics of these biomarkers. Mice were pretreated with XBJ (4 ml/kg) and serum concentrations of IL-6, TNF-α, E-selectin, and vWF were assayed 2, 4, 6, 8, 10, and 12 h after heatstroke. We found that the elevations in all four proteins observed for 12 h after heatstroke were attenuated by XBJ pretreatment, suggesting that the protective role of XBJ in heatstroke was due to its attenuation of circulating pro-inflammatory cytokines and markers of endothelial injury (Figure 3A-D).

### Effects of XBJ on organ injuries

**Figure 4:**
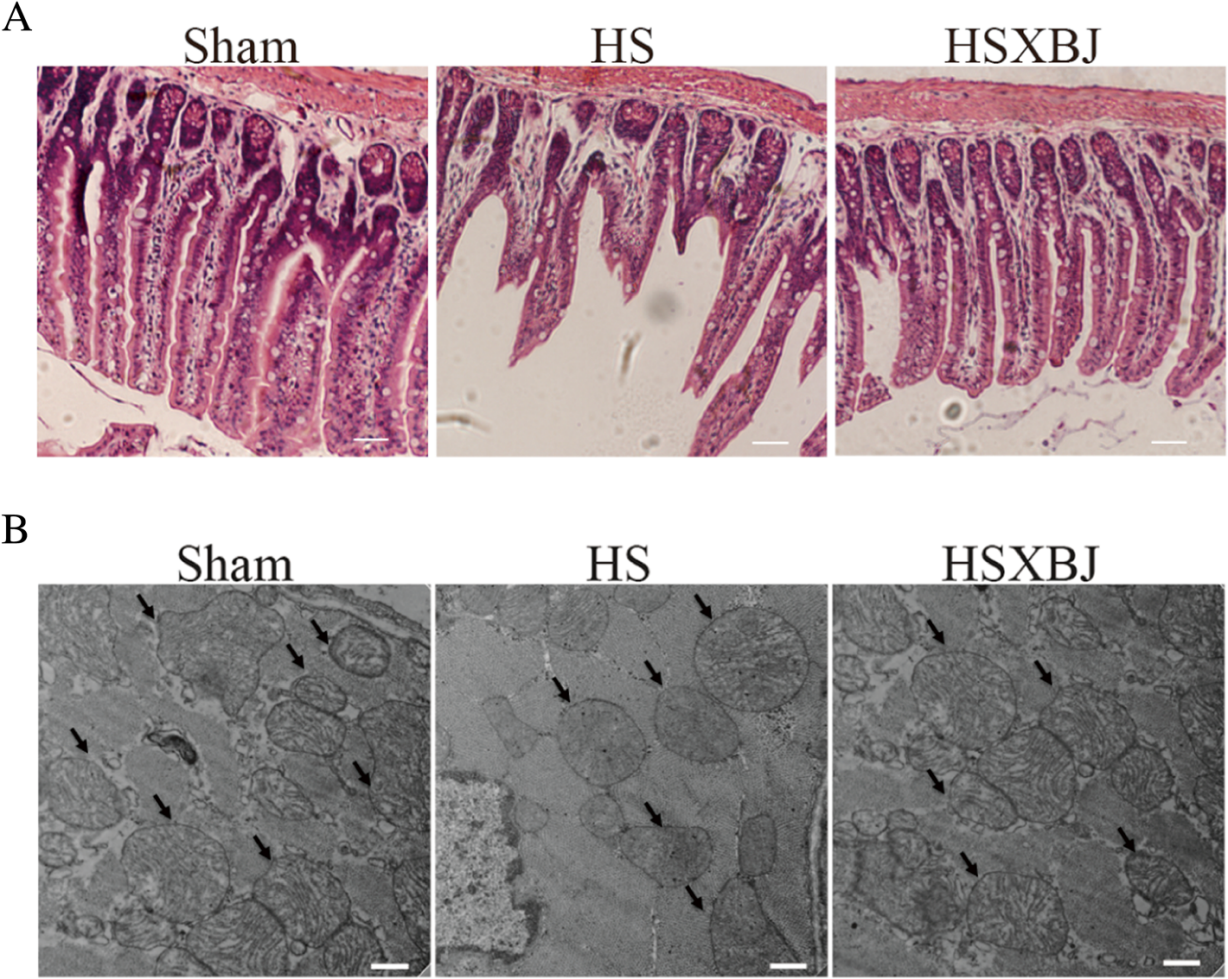
**Histological changes in the jejunum and heart mitochondrial ultrastructure.** Mice were treated with 4 ml/kg of XBJ (HSXBJ group) or 0.9% saline (HS group), followed by heat insults. **(A)** Tissue samples from jejunum were stained with H&E. Scale bar, 10 μm. **(B)** Representative electron micrographs of the heart showing mitochondrial structure (arrows). Scale bar, 500 nm.

To assess the effects of XBJ on organ injuries, mice were sacrificed 6 h after heatstroke onset, and markers of liver, heart and kidney injury, as well as histological changes in the jejunum, were examined. Liver, heart and kidney injuries in heatstroke mice, characterized by markedly elevated serum concentrations of ALT, AST, Cr, BUN, LDH and Tn, were attenuated by XBJ (Table 1). Heatstroke-induced injury to the jejunum, characterized by dramatic shortened villi, disrupted integrity of the intestinal epithelial layer and complete loss of architecture of the lamina propria and submucosa, was diminished by XBJ (Figure 4A).

**Table 1:** **Biochemical markers of tissue and organ injury in mice subjected to heatstroke**

	**Sham**	**HS**	**HSXBJ**
ALT (U/L)	27.0 ± 4.2	283.3 ± 37.9^***^	71.3 ± 10.2^###^
AST (U/L)	21.5 ± 5.4	426.3 ± 47.1^***^	162.5 ± 24.0^###^
Cr (μmol/L)	32.8 ± 6.0	172.0 ± 17.7^***^	116.3 ± 11.5^#^
BUN (mmol/L)	2.9 ± 0.6	18.7 ± 1.4^***^	13.2 ± 1.1^##^
LDH (U/L)	81.0 ± 9.6	439.0 ± 62.7^***^	165.3 ± 22.2^###^
Tn (μg/L)	0.12 ± 0.02	4.08 ± 0.59^***^	1.65 ± 0.31^##^

Mice pretreated with 4 ml/kg of XBJ (HSXBJ group) or 0.9% saline (HS group) were subjected to heat insults. Values represents mean ± SE. ALT, alanine aminotransferase; AST, aspartic aminotransferase; Cr creatinine; BUN, blood urea nitrogen, LDH, lactate dehydrogenase; Tn, troponin. ^***^P < 0.001, vs. sham group; ^#^P < 0.05, ^##^P < 0.01, ^###^P < 0.001, vs. HS group.

### Effects of XBJ on coagulation disturbance

**Figure 5:**
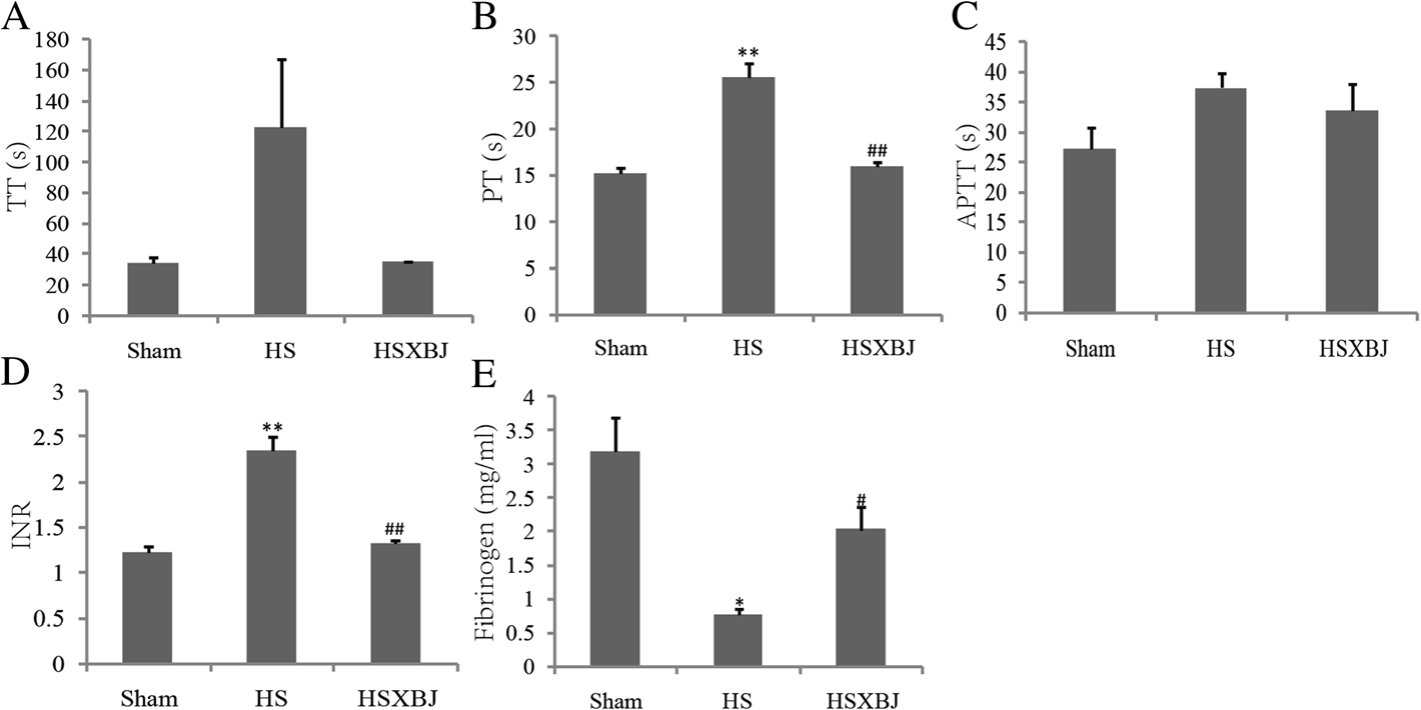
**Changes in coagulation in heatstroke mice.** Mice pretreated with 4 ml/kg of XBJ (HSXBJ group) or 0.9% saline (HS group) were subjected to heat insults. Coagulation was evaluated by measuring **(A)** TT, **(B)** PT, **(C)** APTT, **(D)** INR, and **(E)** fibrinogen. *P < 0.05, **P < 0.001, vs Sham; ^#^P < 0.05, ^##^ *P* <0.001, vs HS group. TT, thrombin time; PT, prothrombin time; APTT, activated partial thromboplastin time; INR, international normalized ratio.

Coagulation disturbance is a marked feature and a leading cause of heatstroke. We evaluated the influences of XBJ on coagulation, and found that HS mice had a significantly prolonged PT, increased INR and elevated serum fibrinogen compared with those in the Sham group. All of these abnormalities, however, were prevented by XBJ (Figure 5A-E).

### Effects of XBJ on mitochondrial structure

As shown by transmission electron microscopy, heat stress caused uniform mitochondrial swelling in the heart, with some mitochondria also showing disorganized cristae, decreased matrix density, and emerging amorphous matrix densities or granular dense bodies. These ultrastructural changes in the mitochondria were diminished by XBJ (Figure 4B).

### Effects of XBJ on mouse survival

**Figure 6:**
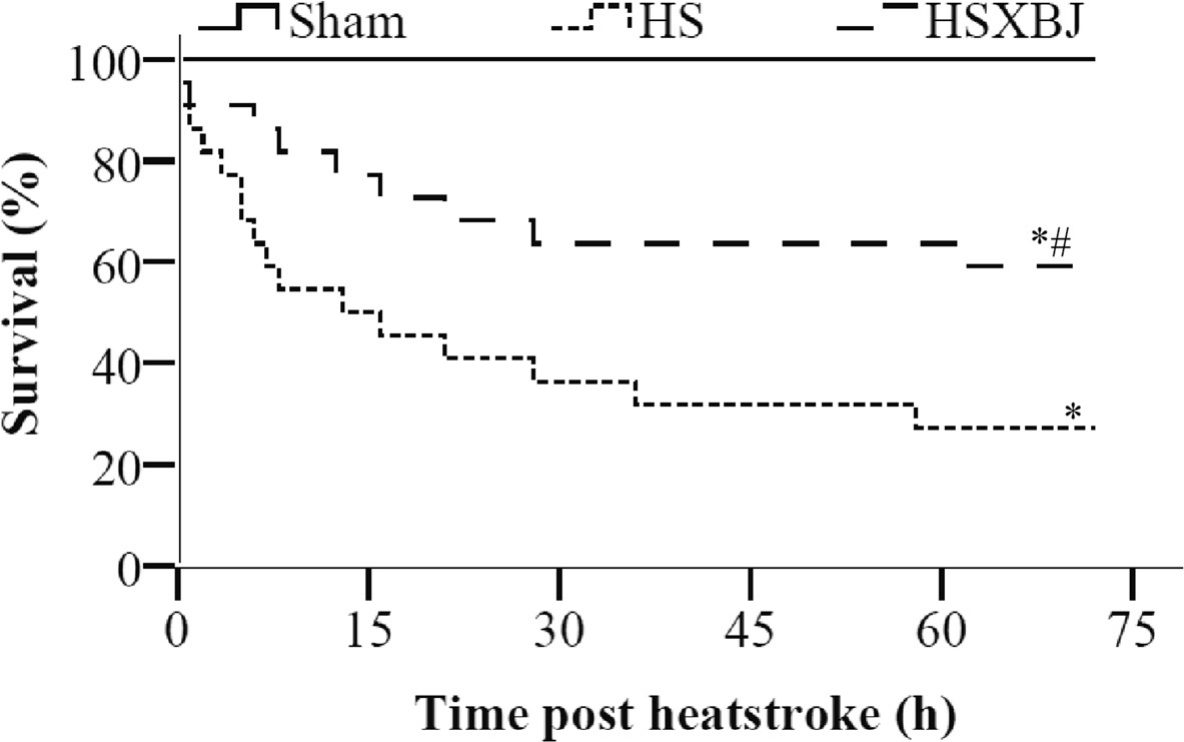
**Effects of XBJ on mouse survival rate.** Mice were pretreated with 4 ml/kg of XBJ (HSXBJ) or 0.9% saline (HS group), followed by heats insult. ^*^P < 0.05, vs Sham; ^#^P < 0.05, vs HS group. sham: n = 8; HS: n = 22; HSXBJ: n = 22.

As our preliminary study showed that no animal died over the 72 h after heatstroke onset, animals in each group were monitored for 72 h to determine their survival rates. All animals in Sham group remained alive, whereas 16 of 22 (73%) in the HS group, and 10 of 22 (45%) in the HSXBJ group died (Figure 6). The survival rate was therefore 38% higher in the HSXBJ than in the HS group (P = 0.006).

## Discussion

Although efforts have been made to develop new methods to treat heatstroke during last decades, few advances have been achieved in early management, except for rapid cooling and prompt fluid resuscitation. Although several drugs have been investigated in the treatment of heatstroke in animals, however, as we know, they are still challenged in patients. XBJ has been found to be effective and safe for the treatment of sepsis and sepsis-related diseases. Most recently, XBJ pretreatment before heat insult was found to reduce plasma concentrations of IL-6 and TNF-α and to decrease organ injuries in rats [20]. However, it is unlikely to administer XBJ before heatstroke in clinical use and since human heatstroke is an emergency condition, which rapidly progresses to MODS and death, it is necessary to assess the effect of XBJ administered after heatstroke onset. Moreover, as a combined traditional Chinese medicine, XBJ possesses other therapeutic effects beyond its anti-inflammation role, which remain to be elucidated. This study showed that XBJ reduced the circulating inflammatory cytokines and markers of endothelial injury, attenuated injuries to the liver, heart and kidneys, restored coagulation balance, and improved survival. Importantly, we found that administration of XBJ before and after heatstroke onset had similar effects. Moreover, we found that XBJ prevents heat stress-induced mitochondrial damage to heart.

Systemic inflammatory response is an important cause of multiple organ injuries in heatstroke. Increased circulating inflammatory cytokines, including IL-6, TNF-α, IL-8 and IL-1β, are commonly observed in animals and patients with heatstroke and high levels of these proteins are correlated with morbidity and mortality [1,11,21,22]. Inhibition of systemic inflammatory responses by electrical vagus nerve stimulation [1], administration of IL-1 receptor antagonist [23], glucocorticoids [24], recombinant activated protein C [9,25], recombinant thrombomodulin [26], or antithrombin [27], or knockout of the gene encoding Toll-like receptor 4, a central regulator of innate immune responses [21], has been found to prevent organ damage and to improve survival. In this study, we demonstrated that XBJ dose-dependently reduced serum IL-6 and TNF-α in heatstroke mice and these anti-inflammation effects lasted for over 12 h. Moreover, XBJ administered before heat insult and after heatstroke onset had comparable anti-inflammation effects.

The mechanism by which XBJ suppresses inflammation remains to be determined. Loss of intestine barrier function is regarded as a fuel of innate SIRS. Prolonged heat exposure decreases splanchnic blood flow and increases nitrosative and oxidative stress, resulting in disruption of tight junctions, loss of intestine barrier function and intestinal hyper-permeability. These, in turn, lead to the translocation into systemic circulation of bacteria and endotoxins normally contained in the gut lumen [28]. In this study, histological examination showed that XBJ maintained the integrity of intestinal barrier structure, preventing the translocation of bacteria and endotoxins and possibly contributing to reduced systemic inflammatory responses. The anti-inflammation activity of XBJ may also be linked to NF-κB, a key transcription factor that controls the induction of pro-inflammatory gene expression and has attracted interest as a new target for the treatment of inflammatory diseases. Recently, some active ingredients in XBJ, including senkyunolide I, safflor yellow A, oxypaeoniflorin, and benzoylpaeoniflorin, were identified to inhibit NF-κB activity by a bio-activity-integrated ultra-performance liquid chromatography quadrupole time-of-flight mass spectrometry assay system [17], indicating that the anti-inflammation activity of XBJ involves inhibition of the NF-κB pathway. Moreover, High-mobility group box 1 (HMGB1), a cytokine mediator of lethal systemic inflammation in sepsis and non-sepsis diseases, has been implicated in heatstroke-induced SIRS and direct inhibition of HMGB1 with its monoclonal antibody can decrease circulating inflammatory cytokines [29]. XBJ can inhibit HMGB1 expression in some sepsis related diseases [14,30], suggesting that XBJ may attenuate inflammatory response partially by down-regulation of HMGB1 expression in heatstroke.

Importantly, our data showed that XBJ reduced serum vWF and E-selectin in heatstroke mice, suggesting a protective role for XBJ in preventing endothelial injury. Endothelium regulates vascular tone, controls leukocyte recruitment, and maintains a balance between pro-coagulant and anticoagulant substances [31]. In heatstroke, widespread microvascular injury, thrombosis and inflammation resulted mainly from endothelial injury contributes to the development of MODS [2,3]. Both vWF and E-selectin synthesized in endothelial cells are elevated in response to inflammation and other stimulations and usually used as molecular markers of endothelial injury. vWF plays a crucial role in platelet adhesion and aggregation, the main initial steps in haemostasis after vascular injury, while E-selectin is important in mediating leukocyte trans-endothelial migration. Moreover, given that cross-talk between endothelial injury, coagulation and inflammation lead to the amplification and exacerbation of inflammatory responses, reduced endothelial injury contributes to ameliorated coagulation disturbances and systemic inflammatory responses in this study. Taken together, our results suggest that XBJ may ameliorate the effects of heatstroke by ablating endothelial injury.

Interestingly, we found that XBJ treatment reduced mitochondrial damages in heatstroke mice. Mitochondria are not only the site of energy production, but also a central locus in the regulation of cell death. Damage to mitochondrial structure generates large amounts of reactive oxygen species (ROS), and triggers cell death pathways of necrosis and apoptosis, which contribute to cell death and tissue injury [32,33]. Therefore, preservation of normal mitochondrial structure is an important cellular mechanism by which XBJ reduces organ injury resulting from heatstroke.

Strikingly, we found that XBJ had similar inhibitory effects on inflammatory responses and endothelial injury when it was administrated before heat insult or immediately after heatstroke onset, with these effects lasting for over 12 h. These findings suggest that XBJ may be used in both the prevention and treatment of heatstroke. However, the effects of delayed administration of XBJ after heatstroke require further investigation.

## Conclusions

In this study, we demonstrated that XBJ reduced organ injury and improved survival in heatstroke mice by attenuating systemic inflammatory responses and endothelial injury, suggesting the potential of XBJ in the prevention and treatment of patients with heatstroke. Although this study was limited to a mouse model and requires confirmation, it provided valuable insights into the role of XBJ in heatstroke. Several other herbal medicines containing anti-inflammation constituents, such as buckwheat sprouts and anti-asthma herbal medicine intervention [34,35], may also protect against heatstroke. It is interesting to evaluate their roles in heatstroke in further studies.

## Abbreviations

XBJ: Xuebijing injuection
vWF: Willebrand factor
IL-6: Interleukin - 6
TNF-α: Tumor necrosis factor - α
IL-1β: Interleukin - 1β
ALT: Alanine aminotransferase
AST: Aspartic aminotransferase
Cr: Creatinine
BUN: Blood urea nitrogen
LDH: Actate dehydrogenase
Tn: Troponin
TT: Thrombin time
PT: Prothrombin time
APTT: Activated partial thromboplastin time
INR: International normalized ratio
ELISA: Enzyme-linked immunosorbent assay
